# Tropical forests in the deep human past

**DOI:** 10.1098/rstb.2020.0500

**Published:** 2022-04-25

**Authors:** Eleanor M. L. Scerri, Patrick Roberts, S. Yoshi Maezumi, Yadvinder Malhi

**Affiliations:** ^1^ Pan-African Evolution Research Group, Max Planck Institute for the Science of Human History, Kahlaische Strasse 10, 07745, Jena, Germany; ^2^ Department of Archaeology, Max Planck Institute for the Science of Human History, Kahlaische Strasse 10, 07745, Jena, Germany; ^3^ Department of Classics and Archaeology, University of Malta, Msida, Malta; ^4^ Department of Prehistoric Archaeology, University of Cologne, 50931 Cologne, Germany; ^5^ School of Social Sciences, University of Queensland, Brisbane, Australia; ^6^ Department of Ecosystem and Landscape Dynamics, Institute for Biodiversity and Ecosystem Dynamics, University of Amsterdam, 1098 XH Amsterdam, The Netherlands; ^7^ Environmental Change Institute, School of Geography and the Environment, University of Oxford, South Parks Road, Oxford OX1 3QY, UK

**Keywords:** tropics, human evolution, tropical forests, rainforest

## Abstract

Since Darwin, studies of human evolution have tended to give primacy to open ‘savannah’ environments as the ecological cradle of our lineage, with dense tropical forests cast as hostile, unfavourable frontiers. These perceptions continue to shape both the geographical context of fieldwork as well as dominant narratives concerning hominin evolution. This paradigm persists despite new, ground-breaking research highlighting the role of tropical forests in the human story. For example, novel research in Africa's rainforests has uncovered archaeological sites dating back into the Pleistocene; genetic studies have revealed very deep human roots in Central and West Africa and in the tropics of Asia and the Pacific; an unprecedented number of coexistent hominin species have now been documented, including *Homo erectus*, the ‘Hobbit’ (*Homo floresiensis*), *Homo luzonensis*, Denisovans, and *Homo sapiens*. Some of the earliest members of our own species to reach South Asia, Southeast Asia, Oceania and the tropical Americas have shown an unexpected rapidity in their adaptation to even some of the more ‘extreme’ tropical settings. This includes the early human manipulation of species and even habitats. This volume builds on these currently disparate threads and, for the first time, draws together a group of interdisciplinary, agenda-setting papers that firmly places a broader spectrum of tropical environments at the heart of the deep human past.

This article is part of the theme issue ‘Tropical forests in the deep human past’.

## 1. The tropics: a frontier for the deep human past

The perception that open grasslands and savannahs were the ecological ‘cradle’ of humans and their ancestors has shaped both the geographical context of fieldwork as well as dominant narratives concerning early hominin evolution, dispersal and cultural development [1,2]. By contrast, tropical forests, where fossil preservation tends to be poorer (e.g. [3,4]), have been presented as relatively pristine environments left free from human influence—habitats deemed too hostile for humans throughout much of prehistory (e.g. [5], see also [6] for overview). Indeed, they have often been framed as the primaeval environments we ‘escaped’ from in Africa, leaving behind the lineages of our close Great Ape relatives [2,7]. These attitudes have profoundly impacted narratives of human evolution in Africa and Out of Africa by introducing enormous biases in the construction of global human prehistory and palaeoenvironments. Such biases have meant that the palaeoanthropological record is fundamentally the human history of a narrow set of habitats, notably along coastlines and in open grassland settings, driving a circular argument that such places are the only areas worth investigating—at the expense of others. These settings and habitats have even been elevated to the status of adaptive cruxes, with ‘savannah corridors’ [8] or coastal ‘highways’ and refugia [9,10] being seen as critical to the cultural efflorescence and dispersal of our species.

**Figure 1.**
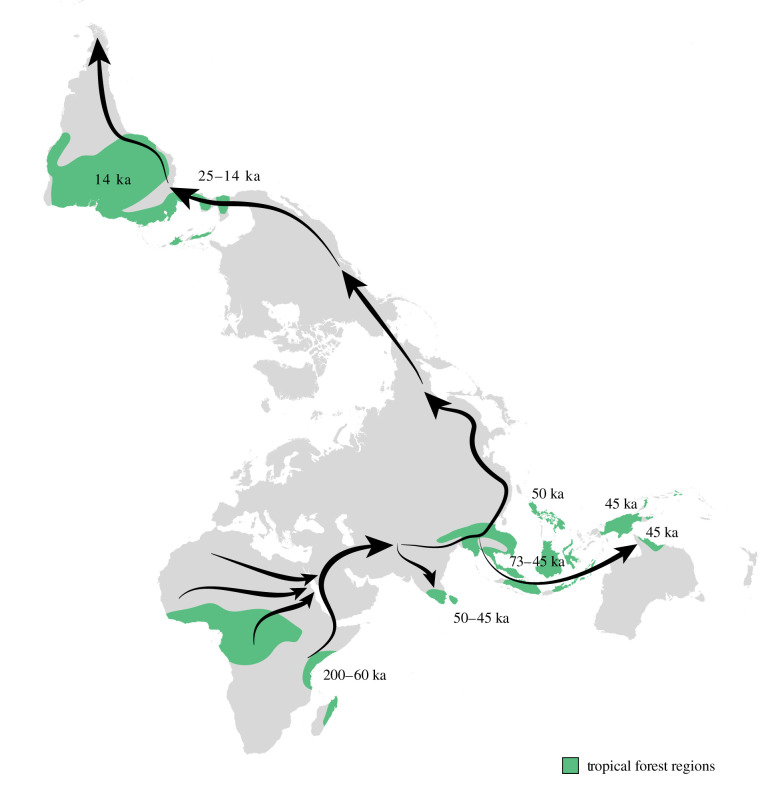
Map of Late Pleistocene human dispersals showing the dates of earliest suggested arrival in the tropical forests of different regions. Green shading shows an artistic approximation of the current tropical forest distribution based on MODIS (moderate resolution imaging spectroradiometer) Land Cover MCD12Q1 majority landcover type 1, class 2 for 2012. Downloaded from the US Geological Survey Earth Resources Observation System (EROS) Data Center (EDC). See Roberts and Petraglia [11].

As *Homo* species spread from Africa, they encountered and engaged with tropical forest biomes across South and Southeast Asia, the Pacific and ultimately, in the case of our own species, the tropical Americas (figure 1). Despite popular perception of vast homogeneous green canopies, the tropical forests of these regions comprise an incredibly diverse set of ecosystems. Although wet, lowland evergreen rainforests are often seen as the classic manifestation of this habitat, ecologists have long noted the huge variety of tropical forests that exist on the planet [12–14]. Semi-evergreen forests with a short annual dry season, montane and sub-alpine forests, closed-canopy dry forests and swamp forests all have different characteristics, structures and species compositions that present a series of challenges and opportunities for hominin populations [15]. In many contexts, tropical forests form mosaic landscapes with open ecosystems such as lowland savannahs or montane grasslands. Furthermore, despite assumptions that tropical forests have been relatively unchanged, there is ample evidence that past fluctuations in precipitation, temperature and CO_2_ concentration have impacted forest form and extent in different parts of the tropics throughout the Miocene, Pliocene, Pleistocene and Holocene [16–18]. As we will also see in this volume, the arrival of hominins, particularly *Homo sapiens*, into these forests may also have introduced further changes to fire dynamics [19–21], species composition [22] and structure [21]. Thus, while tropical forests can be defined as sitting between the latitudes of 23.5° N (the Tropic of Cancer) and 23.5° S (the Tropic of Capricorn), covering the tropics of Central and South America, western and central Africa, western India, Southeast Asia and Oceania, they are far from being homogeneous and, in the case of Australia and China [13], local edaphic and hydrological regimes have led to similar biomes straying beyond the astronomically defined tropics, as they have also done in the past [16]. Some authors refer here to megathermal forests, defined as forest biomes where the risk of frost damage is non-existent, enabling a proliferation of species diversity [16]. In warm periods of Earth history, such as the Eocene, such megathermal forests (functionally tropical forests) have extended to the latitudes of Canada and northern Europe.

Far from being uninhabited by hominins, African tropical forest habitats seem to have been integral to our hominin ancestors [23], and *Homo erectus* notably reached Southeast Asia 1.2 million years ago (Ma), at a time when it has been argued that tropical forest was widespread ([24,25]—although see [26]). These environments likely formed at least part of the backdrop of local trajectories of evolution, as manifested in species such as *Homo floresiensis* and *Homo luzonensis* [27–29]. However, in the history of our genus, it was *Homo sapiens* that went on to most intensively inhabit and exploit tropical forests [6,15]. For many years, this was thought to have been a relatively recent chapter in the human story. Tropical forests were simply considered too hostile. In this view, the dense vegetation, cryptic fauna and sparsely distributed carbohydrates and fats in rainforests made these ecosystems too resource-poor for humans without recourse to sophisticated technologies, external support and exchange systems ([30,31]; see [32]). These views have markedly shaped palaeoanthropological research, particularly in Africa, by focusing fieldwork away from vast swathes of dense forest. Indeed, both ecologically and archaeologically, Africa's tropical forests remain the least well-investigated tropical forests in the world. Although anthropologists, human ecologists and archaeologists have repeatedly reiterated that hunter–gatherers can, and do, permanently live in tropical forests, including rainforests (e.g. discussions in [11,33]), they continue to be frequently neglected in deep time archaeological and palaeoanthropological discussions in Africa.

Instead, it is recent research in Asia that has transformed this field of research by firmly pushing back human exploitation and occupation of tropical forests well into the Pleistocene. Research on the island of Sumatra has found evidence for the presence of humans in rainforests dating to 73 thousand years ago (ka) [34]. In Borneo, a suite of behaviours including the processing of toxic plants, possible alteration of forest edges, and the hunting of forest arboreal fauna has been dated to around 45 ka [35,36]. Seemingly contemporaneously in Sri Lanka, specialist tropical forest adaptations at approximately 45 ka include the hunting of monkeys [37–39], with isotope geochemistry demonstrating a year-round dietary reliance rather than use as seasonal camps [38,40]. These discoveries confirm that intensive exploitation of forest resources has significant antiquity in the human past. Not only that, but they seem to confirm a new, unique ecological adaptability for *H. sapiens* which repeatedly made specialist niche expansions across a broad ecological spectrum well before the beginning of agriculture [41]. Similarly, in South America, humans seem to have occupied tropical lowland and montane forest environments soon after their arrival on the continent (12–14 ka). This appears to have initially taken place along river banks and drier fringes of the lowland and montane forest zone. However, within a few millennia, human occupation pushed deeper into the Amazon forest, primarily along river networks, although archaeological evidence may be biased to such accessible sites [19,20]. Human occupation modes ranged from hunting and gathering to agricultural systems which were based either on locally originated domestications such as manioc and squashes, or imported from Mesoamerica, such as maize.

Despite this growing body of research, however, many major questions remain concerning the deep human past in the global tropics: when did hominins first colonize different tropical forest environments and how did this impact evolutionary trajectories? How did diverse tropical environments drive ancient population structure and the emergence of our species? And finally, when did humans begin to significantly impact and alter tropical forests, and how? This volume draws together a set of state-of-the-art papers investigating these questions from around the global tropics. Starting in Africa, the birthplace of our species, they show that these ecosystems have shaped and been shaped by human agency for millennia. The contributions to this volume also highlight the ways in which diverse, and often novel, methodological applications, from geoarchaeology to isotope analysis, from new chronometric programmes to palaeoecology, are coming together to provide a richer picture of tropical human history.

## 2. African tropical forests

The tropical forests of Africa were the first to be encountered by *H. sapiens* and its hominin ancestors. Africa's forests have particular structural and floral characteristics including an unusually high biomass of animals, which could potentially act as a food resource for humans. Many areas of Africa's humid forests, for example, are sustained by relatively low rainfall that sits at the edge of rainforest viability, which means that even small changes in precipitation can drive dramatic forest fragmentation [17]. Throughout the Pleistocene and Holocene, it appears that many African forests have gone through periods of expansion and contraction as climatic conditions fluctuated, and often a mosaic environment of mixed forests and grasslands was the norm over much of the African tropical forest biome; over the prevailing glacial conditions of the Pleistocene, low humidity and carbon dioxide conditions mean that the overall extent of African forests was generally less than in the present. Tree species diversity in Africa is also lower than in Amazonia and Southeast Asian forests, but taller and larger trees mean that Africa's forests store more carbon than for example, Amazonian forests [42]. These tropical forests also often interdigitate with open grassland regions in a mosaic or patchwork that breaks down a simple dichotomy between open grassland and closed-canopy forest [43]. Such mosaic landscapes may have prevailed over much of the present forest zone throughout the Pleistocene and provided unique, and critical, opportunities for hominins.

The limited current evidence suggests that humans and their ancestors may have been taking advantage of ecotonal regions for a long time. A hominin tooth from Central Africa indicates that at least some early populations were living in mixed environments at the edges of forests around 2.5 Ma [44]. Later on in time, following the emergence of our species, the site of Panga ya Saidi in Kenya shows that humans were exploiting mixed tropical forest/grassland environments *ca* 78 ka, while producing symbolic materials and a variety of technological toolkits [45,46]. If Africa's internal regions hosted the bulk of human populations in the Pleistocene, environments that required humans to flexibly shift between diverse ecotones may have formed the cradle for our species' ecological modernity. In this emerging view, the reliance on different resources may have been the driver that set populations apart, rather than the environments themselves (e.g. [47]). These processes may sit at the root of our species, which is now thought to have evolved in subdivided populations across much of the continent [48]. When did this, and a hominin focus on tropical forest occupation, begin?

Braucher *et al*. [49] suggest a longer history than previously supposed. They report the oldest evidence of a hominin presence in the Congo Basin, with a minimum age of between 850 and 650 ka. Discovered in 1987, Elarmékora is a high terrace sitting above the Ogooué River within the Lopé National Park in Gabon. The authors present the first absolute dates for the small lithic assemblage found there, including mainly cobble artefacts embedded within alluvial material. Cosmogenic nuclide assessments suggest a minimum age of between 730 and 620 ka for the undiagnostic Earlier Stone Age assemblage. This age is among the oldest documenting a hominin presence in western Central Africa and confirms the long legacy of hominins in this region. These results indicate that the long-held assumption that a hominin presence in tropical forests only emerged following the arrival of agriculture should be rejected, and reorients geographical assessments of human dispersals in and beyond Africa.

This tantalizing picture of a long-term hominin presence in the tropical forest regions of Africa sits within a backdrop of 1 million years of dynamic climatic and environmental change. Here, Gosling and colleagues [50] synthesize information on Pleistocene and Holocene vegetation changes from long-term terrestrial and marine records, showing how the locations of vegetative resources for hominins shifted geographically over time (see also [51]). Of particular interest is the fact that the hominin presence in the Congo Basin described by Braucher *et al*. [49], coincides with generally humid conditions and therefore likely a period of forest expansion, rather than fragmentation. Furthermore, a profound shift in the hydro-climate in the last 1 Myr in Africa, leading to eastern and western parts of the forest zones being alternately wetter and drier, occurs at a time when the first fossil appearances of our species have been suggested elsewhere in Africa (e.g. [52]). For later time periods associated with *H. sapiens*, vegetative changes were clearly asynchronous in different regions, likely producing the conditions for mixed resource acquisition in many regions and necessitating adaptability.

Taylor [53] specifically pursues the question of mixed resource acquisition through Pleistocene material culture from the Middle Stone Age (MSA), the first and longest-lasting technological repertoire associated with our species. Specifically, he looks at the Lupemban, a stone tool (lithic) technocomplex that has long been associated with Africa's equatorial forests at the site of Kalambo Falls in Zambia. Here, the Lupemban has been best dated to between 270 and 170 ka. Today, Kalambo Falls is dominated by Miombo woodland, and Twin Rivers, another key Lupemban site, by open woodland-bushland. While both sites are just beyond current areas of forest, they may have been within forest zones in the past. Given the frequent interdigitation of open and closed environments in Africa's forests, Taylor argues that *H. sapiens* may have been adopting a flexible strategy within ecotonal areas that may indicate a partial reliance on forest resources. Taylor concludes that the lanceolate points of the Lupemban may have presented an adaptation to a vegetation mosaic that underscores a potentially unique human niche.

These results complement the work from Blinkhorn *et al*. [54] on the availability of refugia in tropical Africa. Refugia are places that remained stable and habitable through various cycles of climate change (see [55]). As the only continent where *H. sapiens* have clearly persisted through multiple glacial-interglacial cycles, Africa is a key area where classic refugia models can be formulated and tested. Blinkhorn *et al*. [54] apply climatic thresholds on human habitation, rooted in ethnographic studies, in combination with high-resolution model datasets for precipitation and biome distributions to identify persistent refugia spanning the Late Pleistocene (130–10 ka). Remarkably, Blinkhorn and colleagues find that refugia were unlikely to be rare phenomena during the Late Pleistocene, even using conservative estimates. One region that emerges as among the most stable is the modern-day Sene-Gambia region, where MSA assemblages have been remarkably persistent [47,56]. Blinkhorn and colleagues also highlight the broad distributions of stable ecotonal areas, which may have been critical for long-term human habitation [45,51,57].

Moving on in time, Orijemie [58] synthesizes past climatic variability in the forest of West-Central Africa during the Late Pleistocene–Holocene period to understand the interaction of climate on the development and stability of human communities in the region over time. Combining palaeoclimate and vegetation histories, Orijemie highlights the significance of climate variability on the development and survival of early hominin ancestors and humans in the forest regions of West-Central Africa. In response to major climatic fluctuations, West-Central African savannahs expanded at the expense of forests, but did not transit into strictly ‘forest’ or ‘savannah’ blocks. Rather, the forests had a variety of vegetation types and biodiversity ecotones, even during periods of environmental stress. These data suggest heterogeneous and resilient forest ecosystems. Human behaviours exhibited in the form of technological modifications and changes in subsistence strategies, varied independently of climate and vegetation changes, suggesting climate was not the prevailing driver of human behaviour or community stability.

This brings us to the present day, and Boyette and colleagues synthesize genetic, paleoclimatological, and historical linguistic data on the peopling of the Congo Basin and use this to build on their ethnographic work in the northern Republic of Congo with BaYaka foragers living along the Motaba river. They argue that the cultivation of ‘relational wealth’, that is, the forming of strong social ties to enable exchanges of resources and mutual assistance, is key to living in tropical forest environments. This currently includes the cultivation of such wealth among different forest forager groups as well as trading relationships with farmers. Here, Boyette and colleagues argue that it is a mistake to cast this trading as a dependence of foragers on farmers. The BaYaka are seasonally mobile with their own forest gardens, created using knowledge learned from farmers, as well as the creation of spaces for the growth of wild foods such as *Dioscorea* yams. They are also highly seasonally mobile, with some 82 km being the largest distance between where a parent was born and where their adult child now lives. Indeed, Boyette and colleagues argue that mobility is central to the flow of knowledge throughout the Congo Basin, including subsistence innovations and forest spirit dances. This complements the work of the previous papers that indicate that a high degree of mobility was always required to successfully live in this region. At the same time, Boyette and colleagues review the genetic studies that indicate that western and eastern branches of the forager populations split between 30 and 20 ka, probably following forest fragmentation well before the beginning of agriculture. This implies that significant breaks between different ecosystems may have been major boundaries in the past to populations either adapted to mixed resources or specific habitats.

## 3. Southeast Asian and pacific forests

Since no continuous tropical forest belt exists between the African and southern and eastern Asian forests (figure 1), moving into other parts of the tropics must have involved repeated adaptation to varied tropical forest ecosystems. In fact, human groups expanding beyond Africa would have encountered significantly drier landscapes that spread into the Thar Desert of India before re-entering tropical zones again [59–61]. Once encountered, the Asian tropical forests presented a completely different set of floral and faunal characteristics compared to those in Africa. In contrast with Africa, Asian tropical forest extent was probably greater throughout the prevailing glacial conditions of the Pleistocene, as low sea levels greatly increased land area and connectivity in Sundaland and Sahul, while the generally maritime climate maintained high rainfall [62]. Moving into the tropical forests of Wallacea and the Pacific, humans would also have to contend with unique insular tropical ecosystems and the necessity of seafaring (see [63]).

It is in Asian tropical forests that archaeological and palaeoanthropological evidence began to highlight the critical role of tropical forests in early human adaptations and dispersals. Be it in Sumatra 73 ka [34], Borneo 50–45 ka [35,36], Sri Lanka 45 ka [37,39,64] and perhaps also southern China as early as 100 ka [65], human populations appear to have repeatedly adapted to tropical forest environments rapidly following their arrival in different parts of tropical Asia. These adaptations do not correspond to a constant wave, with uniform technologies, but rather highlight repeated, variable responses to different forest settings. For example, findings of the bow and arrow and clothing manufacture in Sri Lanka 45 ka [66] provide a very different context for this innovation than assumptions of its association with drying grasslands or European tundra conditions. Similarly, although the ‘Hoabhinian’ core and flake technologies found across much of Southeast Asia during the Late Pleistocene had been previously considered ‘simple’, more recent work and experimental analyses have highlighted the potential flexibility of these stone tools and their likely association with the manufacture of organic artefacts [67].

Understanding the exact context of human arrival in Southeast Asia has been plagued by issues of site and artefact preservation, correlation between hominin and palaeontological records, as well as issues with chronology construction. In this volume, Louys *et al*. [68] re-examine the fossil deposits of Lida Ajer in Sumatra which documents some of the earliest evidence for the presence of modern humans in tropical forests. Two human teeth from this cave were estimated to be 73–63 kyr old, which is significantly older than estimates of modern human migration out of Africa based on genetic data. The authors provide a new assessment of the available ages and stratigraphic information from the site, confirming its antiquity. The deposits were previously interpreted as rainforest based largely on the presence of abundant orangutan fossils, although their exact ecological preferences remained debatable. The use of stable carbon and oxygen stable isotope analyses of mammalian fossil tooth enamel further demonstrates that early humans likely occupied the site during marine isotope stage 4 (MIS 4; *ca* 74–60 ka) dominated by a closed-canopy forest very similar to those present in the region today, although the fossil orangutans appear to have occupied a slightly different niche in the rainforest than their modern counterparts.

Similarly, McAdams *et al*. [69] undertake geoarchaeological analysis of two archaeological cave sites in Vietnam. By MIS 3, it is clear that our species had dispersed throughout much of Southeast Asia, including the diverse forest systems of upland Vietnam. Here, wetter, sheltered conditions resulted in forest refugia that were attractive to early human populations, with the collection of diverse resources, such as land snails, providing resilience subsistence strategies. Nevertheless, the middens which record such evidence, and the caves in which they are formed, are subject to a series of unique diagenetic and site formation processes that need to be better understood to understand the nature and tempo of human adaptations and settlement patterns. McAdams and colleagues show how thin-section micromorphology is providing more refined insights into depositional and post-depositional sites across tropical zones, providing a basis for wider analysis of our species' interaction with tropical forests around the world.

Finally, moving out into the Pacific realm, Roberts *et al*. [63] present new radiocarbon and stable isotope data from the earliest human remains so far excavated in tropical island settings in Near and Remote Oceania. This is a key region for exploring early maritime crossings, human adaptations to insular and coastal environments, and the possibility of interactions between different hominin species. Roberts *et al*. [63] show that there is currently a significant gap between the earliest occupation of the portion of Near Oceania beyond the continent of Sahul approximately 45 ka and the oldest human remains from the region approximately 11.8 ka. However, the authors demonstrate that Late Pleistocene–Holocene humans living on islands in the Bismarck Archipelago and Vanuatu had a persistent reliance on tropical forest plants and animals. These habitats, rather than solely coastal settings and arriving domesticates, provided critical settings for human adaptation and landscape manipulation.

## 4. Neotropical forests

Current archaeological and genomic data suggest that the Americas were colonized sometime between approximately 25 and 15 ka by modern humans likely following the Pacific Rim corridor from northeast Asia into the New World, reaching southern Chile by *ca* 14.3 ka [70–72]. Early human populations in the Americas have traditionally been portrayed as mobile hunter–gatherers who exploited coastal resources and large savannah game, while avoiding forest habitats as a result of the absence of large mammals and the difficulties of mobility in dense forest vegetation [73–75]. Contrary to this classic paradigm, mounting evidence suggests early colonists were actively exploiting and managing trees of economic importance and quite quickly began practicing early cultivation of annual crops [76–83]. These data have important implications for understanding plant domestication, the long-term legacy of human–plant interactions and the potential role of humans in the current hyperdominance of useful plants in Amazonia [22,84,85].

In this volume, Bush *et al*. [19] and Nascimento *et al*. [20] synthesize paleoecological data to paint detailed pictures of the timing and ecological impacts of early human arrival in the tropical Andes and Amazon lowlands, respectively. In the Andes, the earliest evidence of human occupation occurs around 14–12 ka, coinciding with a time of rapid climate change as species were migrating upslope in response to deglacial warming. The retreat of the glaciers opened up the relatively flat and dry areas of the upper montane Andes (3000–4000 m elevation), and this region seems to have been among the most amenable American tropical regions for first human settlement (see also [86]). By 12 ka most areas now characterized as high elevation Andean grasslands (*puna* and *paramo*) were being burned and modified. Bush and colleagues suggest these extensive grasslands should be regarded as long-term anthropogenic Holocene landscapes, and likewise the sharp treeline between the forests of the Andean flank and the grasslands should be regarded as anthropogenic rather than climate-defined. These dense forests of the montane flank were probably less settled than the flatter and drier upland regions for both topographic and climate reasons, though by the mid-Holocene accessible regions of the montane forest zone were substantially modified and settled [19].

In the extensive Amazon lowlands, the first evidence of human occupation appears around 12 ka, located mainly along the Amazon river and the dry forest-savannah mosaic of the Amazon forest periphery. The more forested areas of southern Amazonia show signs of occupation from 6 ka, with substantial increase in range and density since 4 ka. By the time of European arrival, human occupation had spread across much of the Amazon biome, particularly along its river networks. The earliest human settlers of the Americas encountered continents rich in exotic and now-extinct megafauna, and this is likely true of the tropical Americas as much as for high latitudes. Overall, 34 out of 47 megafaunal species became extinct in South America [87,88]. These megafauna were undoubtedly in the savannah, Andean grassland and savannah-forest transition zones, but the direct evidence of megafaunal occupation of the dense forest zone (as occurs, for example, in African tropical forests) is limited and hampered by poor preservation. The direct cause of the extinction seems to be a confluence of rapid climate change putting wildlife populations under stress, coupled with human pressures through hunting and habitat modification adding additional pressure and preventing the recovery from refugia that occurred after previous periods of environmental variability.

By examining paleoecological evidence from lakes across the Andes, Bush *et al*. [19] describe the timing of this transition, with widespread demise of megafauna around 12.5 ka, soon after an increase of fire. They propose the megafauna were stressed by the rapid warming and wet conditions of the deglaciation and population recovery was prevented by hunters who transformed the high Andean landscape through burning. Iriarte *et al*. [89] present a compelling picture of this first encounter between Neotropical humans and megafauna, making a detailed case based on rock art found at Serranía de la Lindosa, Colombia, on the present-day ecotone between the northwestern Amazon forest and the Orinoco savannahs. They suggest that this art dates from the Late Pleistocene (around 12.6 ka) and among many other things depicts lost megafauna such as giant sloth (probably *Eremotherium*), a camelid (possibly *Paleollama*) and a three-toed ungulate (probably *Xenorhinotherium*).

Human impacts on Neotropical forests also involved interaction with plant communities [90], and the region is home to the smallest temporal gap between human arrival and cultivation practices in the tropics. An independent Amazonian origin of agriculture has been a particularly significant discovery in recent years, with manioc (*Manihot*) and squash (*Cucurbita*) cultivation appearing on artificial forest islands in the seasonally flooded savannahs of Beni, Bolivia as early as 10.4 ka [78]. Cultivation dating to 9 ka also appears in the forest zone north of the savannahs [91], and there are signs of cultivation near campsites in northwest Amazonia [80]. In regions away from plant cultivation, early- to mid-Holocene foragers consumed palms, tree fruits and nuts [20]; many of these species are now hyperdominant in Amazonia and it has been suggested that the elevated abundance of these species across Amazonia may reflect selection and stewardship by indigenous populations over millennia [84].

The extent to which Amazonia is a cultural landscape with a significant long-term human footprint is still disputed, however [19]. Nascimento *et al*. [20] present an extensive paleoecological synthesis of the ecological effects of early human occupation of Amazonia. Significant vegetation changes are often argued to be found only centuries to millennia after the first signs of human settlement in forests, suggesting that the earliest occupants exerted only a gradual change on the forest. The dry forest-savannah zone seems to have been particularly favoured; as in Africa, this mosaic landscape provides a wide range of resources, and also the possibility of working with and enhancing natural fire regimes to aid vegetation clearance and ecosystem transformation. Maezumi *et al*. [21] examine the role of land use, cultural burning and soil enrichment in shaping the composition and structure of the Amazon forest ecotone. They integrate 6000 years of archaeological and palaeoecological data from Laguna Versalles, Bolivia which was dominated by stable forest vegetation throughout the last 10 000 years. These data document the management of forest composition and structure, cultural burning, cultivation of edible plants and the formation of anthropogenic Amazonian Dark (ADE) soils. Frequent cultural burning altered ADE forest composition and structure by controlling ignitions, decreasing fuel loads and increasing the abundance of fire-adapted plants.

With the expanding and varied record of human history in Neotropical forests now established, it remains to explore how human occupation of the varied habitats available, from seasonally dry forests to lowland rainforests, impacted patterns of human settlement, adaptation and culture. In this volume, Sales and colleagues use a statistical approach to explore the spatial distribution of Indigenous populations across the tropical Andes prior to European arrival. They note how variability in elevation, cloud frequency, river proximity and seasonal aridity may have significantly shaped human occupancy. Sales and colleagues present an estimate of the portion of this area occupied by pre-Columbian populations and note how a number of forest ecosystems still document anthropogenic influence centuries later. Further detailed investigations of the tropical forests of the Andes, and elsewhere in tropical North, Central and South America should enable a more detailed understanding as to the tempo and nature of repeated human adaptations to the tropical forests of this region through the Pleistocene and Holocene.

## 5. Synthesis

**Figure 2.**
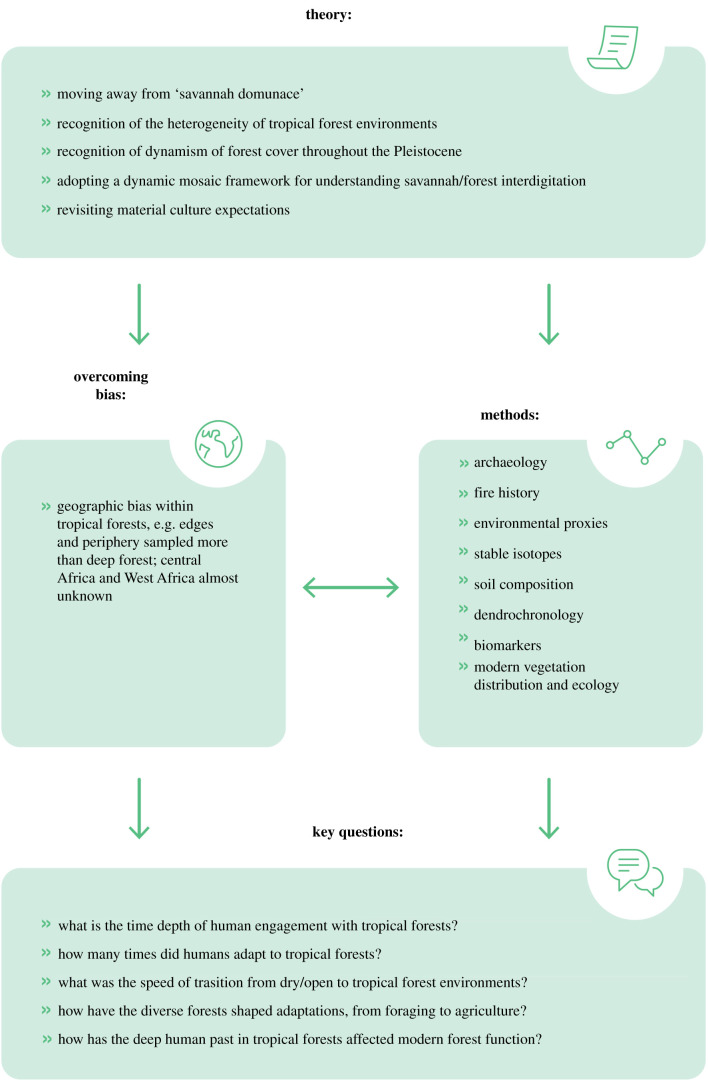
The relationship between theory and research goals for understanding the role of tropical forests in the deep human past. (Online version in colour.)

Tropical forests clearly represent a key human habitat that can no longer be ignored in the context of deep human history. In particular, the wealth of data, methods and insights emerging from tropical forests in Asia and South America is driving a tropical research agenda that has so far lagged somewhat behind in Africa, the evolutionary home of our species. What can be said so far, and what are the major future research questions and approaches (figure 2)?

Perhaps the most obvious outcome of increasing archaeological research in tropical forests is that we can no longer afford to think about them as peripheral areas to the main stage of human evolution and the early human past. Despite the persistence of various hypotheses tied to savannahs, grasslands and coasts across both the Old and the New World, humans are fundamentally plastic in their behaviour [92]. This plasticity is seen among earlier *Homo* species, as well as our own. As an extreme example, it is remarkable how humans adapted from being Arctic hunter–gatherers to Amazonian cultivators within a few millennia. It therefore seems unlikely that humans ever restricted themselves to any single narrow set of resources [41]. Indeed, it seems unlikely that the pan-African distribution of the MSA—the earliest and longest-lasting cultural phase associated with our species—was only ever present in grasslands and savannahs. Building on this, researchers must begin to abandon simple dichotomies between ‘rainforest’ and ‘savannah’ as mutually exclusive areas of human habitation.

Along a spectrum of adaptation, it may well be that various human groups found specialist solutions to their particular habitat of choice; however, in many cases specialization is likely found in the ability to remain flexible and exploit a range of habitats and their resources [41]. Indeed, it is the clear, repeated ability of our species to adapt in different ways to these habitats, among others, that might be what sets us apart from our closest relatives. As we have seen, for example in Africa, tropical forests are not themselves homogeneous blocks. Instead, forests for example, can interdigitate with clearings, drier forest types, palm swamps, gallery forests, grassy floodplains and savannahs that invite such flexible exploitation. To investigate this further, it seems clear that vast swathes of tropical forests remain to be investigated. Despite the emerging work in Southeast Asia and Amazonia, substantial areas remain near completely unexplored, particularly in Africa, for what they can say about the deep human past. What expectations should we have, and what methods should we be using?

The papers of this volume also highlight that many of the most recent advances in our understanding of early human encounters with tropical forests have involved the application of varied methodologies that cut across the social and natural sciences. Resolving the role of tropical forests in the deep human past is clearly a truly interdisciplinary endeavour, often involving ‘an archaeology of the invisible’. For example, traces of human activities may be found in the current distributions and community composition of wild plants and trees (such as palm nuts in the Congo Basin and brazil nuts in the Amazon Basin), in patterns of charcoal accumulation [93] and in alterations of soil composition in palaeoenvironmental cores and archaeological sites [83], and faunal communities [39] (figure 2). The study of the growth rings of living trees (dendrochronology) has even been shown to track human management of forests in more recent periods [94]. In warm and wet ecosystems where organic preservation is low and sites difficult to find and locate, the traces of human impact on the environment may sometimes be the only evidence of past occupation. Stable isotope analysis of human tooth enamel has also emerged as a means of assessing overall dietary reliance in the face of incomplete plant and animal assemblages [38,40,63]. Such sensitive approaches must be combined with traditional archaeological investigations in order to fully appreciate the context of past human engagement with tropical forests.

**Figure 3.**
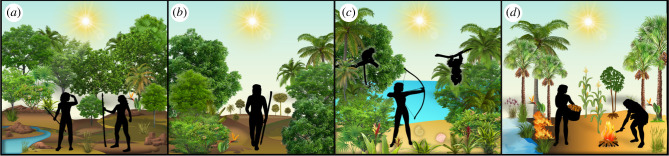
Conceptual figure of land use in: (*a*) hominins using the forest edge, (*b*) early humans exploiting forest resources, (*c*) specialized adaptations in the forests of Sri Lanka/South Asia and (*d*) polyculture agroforestry in Amazonia. (Online version in colour.)

When it comes to the archaeology of Pleistocene tropical forests, we should not necessarily always expect radically transformed stone tool types, but also more generalist and flexible tools capable of dealing with a dynamic contextual environment (figure 3). In Africa, regionalization of the MSA may shed more light on the degree of isolation between groups rather than purely environmental determinants, and clearly a range of MSA tools can be used in a wide variety of contexts. Ubiquitous, generic elements of MSA toolkits are also found across Africa for over 300 thousand years, suggesting they met flexible and dynamic needs in a variety of environments. Indeed, examples from the rainforests of Southeast Asia suggest that specialist adaptations can be found beyond simply lithics, in the form of the development of organic tools involving bamboo and other materials, in the type of prey targeted, in possible trapping techniques that may leave no trace, and in the treatment of carbohydrates such as the detoxification of tubers [95]. Southeast Asian ‘Hoabhinian’ technologies (see [96]) may provide an interesting comparison to MSA technologies in West and Central Africa in future, in this regard, although lithics analyses have often retained a local and regional focus. Meanwhile, microliths and bone tools found in Sri Lanka, argued to be part of early bow and arrow technologies [66], indicate another route towards specialized tropical adaptation.

When it comes to unravelling this global tropical record of human evolution, the unknowns are numerous. Despite the work done to date, we still often have no clear ideas of when *H. sapiens* first began to intensively exploit different types of tropical forests in a given region, and whether such behaviour can also be observed in ancestral species (figure 3). We also do not know how this may have specifically been characterized. Did past Pleistocene forest foragers rely on high mobility and strong social networks? How did they navigate the forest, for example, using forest elephant trails in Africa, as well as the river networks that may have been key for human mobility in Amazonia? How may they have used the forest seasonally, for example, by controlling the distribution and location of preferred wild foods at certain times of year, such as the land snails of North Vietnam? Many of these questions have long been asked of Pleistocene sites in temperate Eurasia and southern and eastern Africa. However, a general absence of tropical forests in wider theoretical discussions in human evolution means these themes are only just starting to become accessible for these environments. At a broader level, it is still unclear whether tropical forests could sometimes still represent significant barriers, for example, driving population structure. How important were forest edges and ecotonal regions in human evolution?

Moving into the Holocene, the evidence is a little less sparse, but many questions remain. Many of the biases can also still be found. For example, research in forests continues to be dominated by geographic biases, for example, focusing on rivers or dry margins. In Africa, forest research in the Holocene still lags behind similar work in the Americas in particular. Yet the Late Pleistocene and Holocene also provide opportunities through which human adaptations to forests can be better understood, as there are multiple cases of human colonization of tropical forest environments that can be compared and contrasted. How was the ecology of the tropical forests that humans occupied in the Late Pleistocene and early Holocene different from those of the late Holocene, given changing atmospheric carbon dioxide and dynamic shifting mosaic landscapes? How did megafauna either hinder or facilitate forest occupation? How do biogeographical differences across the tropics, such as the relatively low-fruit abundance in the wind-dispersal-dominated dipterocarp forests of southeast Asia, affect how early humans used forest resources? And how may the long history of human occupation of these forests have also shaped the species composition of modern-day tropical forests?

The ‘big questions’ that remain are summarized in box 1. Addressing these will require the continuation and expansion of foundational research across the global tropics, alongside the recognition that there is a whole spectrum of tropical forest habitats, not just ‘rainforests’. The pursuit of these goals will require the investment of funding agencies and a commitment of risk to further research. These are, after all, not the ‘well-trodden’ regions of grassland and savannah, where a wealth of previous discoveries robustly attests to future potential. In particular, funding for local researchers to lead multidisciplinary investigation of tropical regions will be essential to boosting tropical forest archaeological and palaeoanthropological research. Investment in tropical forests, and researchers within the tropics, will lead to a new and enriched understanding of the deep human past: the accumulation as well as the importance of evidence to date unmistakably supports this view. This volume represents a substantial step in furthering this goal and represents a call to scholars and funders alike to give new attention to how our collective human prehistory interweaves with this globally important ecological region.

Box 1. **Big Questions.**

1. What is the time depth of human and even hominin engagement with tropical forests?
2. How many times did humans adapt to tropical forests?
3. How do repeated adaptations to tropical forests compare across the global tropical belt, and are they underpinned by any commonalities specific to these environments?
4. What was the speed of transition from dry/open to tropical forest environments, and how did forest-savannah transition zones act as entry points?
5. How can we characterize the dynamism of tropical forest climate and distribution throughout the Pleistocene, and which were the mosaics favoured by early humans?
6. Were dense tropical forests largely barriers or corridors?
7. How does the varying ecological biogeography of tropical forests affect how they have been used and stewarded by humans in the past?
8. Do mosaic forest environments generate new resources greater than the sum of forest and savannahs alone (e.g. edge specialist species)?
9. How have the diverse forests shaped adaptations, from foraging to agriculture, e.g. discussions of seasonal environments as critical to early tropical cultivation? How far does this hold, and what were the legacies of diminishing megafauna?
10. How has the long history of human interaction with tropical forests influenced the modern ecology and function of these forests?

## Data Availability

This article has no additional data.

## References

[1] Dennell C, Roebroeks W. 2005 An Asian perspective on early human dispersal from Africa. Nature **438**, 1099-1104. (10.1038/nature04259) 16371999

[2] Domínguez-Rodrigo M. 2014 Is the ‘Savanna Hypothesis’ a dead concept for explaining the emergence of the earliest hominins? Curr. Anthropol. **55**, 59-81. (10.1086/674530)

[3] Tappen M. 1994 Bone weathering in the tropical rain forest. J. Arch. Sci. **21**, 667-673. (10.1006/jasc.1994.1066)

[4] Mercader J, Runge F, Vrydaghs L, Doutrelepont H, Ewango C, Juan-Tresseras J. 2000 Phytoliths from archaeological sites in the tropical forest of Ituri, Democratic Republic of Congo. Quat. Res. **54**, 102-112. (10.1006/qres.2000.2150)

[5] Meggers BJ. 1954 Environmental limitation on the development of culture. Am. Anthropol. New Series **56**, 801-824. (10.1525/aa.1954.56.5.02a00060)

[6] Roberts P. 2021 Jungle: how tropical forests shaped the world – and us. London, UK: Viking Books.

[7] Darwin CR. 1871 The descent of man, vol. 1. London, UK: John Murray.

[8] Bird M, Taylor D, Hunt C. 2005 Palaeoenvironments of insular Southeast Asia during the last glacial period: a savanna corridor in Sundaland? Quat. Sci. Rev. **24**, 2228-2242. (10.1016/j.quascirev.2005.04.004)

[9] Mellars P. 2006 Why did modern human populations disperse from Africa *ca.* 60,000 years ago? A new model. Proc. Natl Acad. Sci. USA **103**, 9381-9386. (10.1073/pnas.0510792103) 16772383 PMC1480416

[10] Marean C. 2010 Pinnacle Point Cave 13B (Western Cape Province, South Africa) in context: the Cape Floral kingdom, shellfish, and modern human origins. J. Hum. Evol. **59**, 425-443. (10.1016/j.jhevol.2010.07.011) 20934095

[11] Roberts P, Petraglia MD. 2015 Pleistocene rainforests: barriers or attractive environments for early human foragers? World Archaeol. **47**, 718-739. (10.1080/00438243.2015.1073119)

[12] Wilson EO. 1988 The current state of biological diversity. In Biodiversity (ed. EO Wilson), pp. 3-18. Washington, DC: Washington National Academic Press.

[13] Whitmore, TC. 1998. An introduction to tropical rainforests, 2nd edn. Oxford, UK: Oxford University Press.

[14] Malhi Y. 2010 The carbon balance of tropical forest regions, 1990–2005. Curr. Opin. Environ. Sustain. **2**, 237-244. (10.1016/j.cosust.2010.08.002)

[15] Roberts P. 2019 Tropical forests in prehistory, history and modernity. Oxford, UK: Oxford University Press.

[16] Morley RJ. 2000 Origin and evolution of tropical rain forests. New York, NY: John Wiley & Sons.

[17] Malhi Y, Wright J. 2004 Spatial patterns and recent trends in the climate of tropical rainforest regions. Phil. Trans. R. Soc. B **359**, 311-329. (10.1098/rstb.2003.1433) 15212087 PMC1693325

[18] Bush M, Hanselman JA, Hooghiemstra H. 2007 Andean montane forests and climate change. In Tropical rainforest responses to climatic change (eds M Bush, J Flenley, W Gosling), pp. 35-60. Berlin, Germany: Springer.

[19] Bush MB, Rozas-Davila A, Raczka M, Nascimento M, Valencia B, Sales RK, McMichael CNH, Gosling WD. 2022 A palaeoecological perspective on the transformation of the tropical Andes by early human activity. Phil. Trans. R. Soc. B **377**, 20200497. (10.1098/rstb.2020.0497) 35249394 PMC8899620

[20] Nascimento MN, Heijink BM, Bush MB, Gosling WD, McMichael CNH. 2022 Early to mid-Holocene human activity exerted gradual influences on Amazonian forest vegetation. Phil. Trans. R. Soc. B **377**, 20200498. (10.1098/rstb.2020.0498) 35249380 PMC8899618

[21] Maezumi SY et al. 2022 Legacies of Indigenous land use and cultural burning in the Bolivian Amazon rainforest ecotone. Phil. Trans. R. Soc. B **377**, 20200499. (10.1098/rstb.2020.0499) 35249381 PMC8899619

[22] Ter Steege H et al. 2013 Hyperdominance in the Amazonian tree flora. Science **342**, 1243092. (10.1126/science.1243092) 24136971

[23] White TD, Asfaw B, Beyene Y, Haile-Selassie Y, Lovejoy CO, Suwa G, WoldeGabriel G. 2009 *Ardipithecus ramidus* and the paleobiology of early hominids. Science **326**, 64-86. (10.1126/science.1175802) 19810190

[24] Sémah F, Sémah AM, Simanjuntak T. 2002 More than a million years of human occupation in insular Southeast Asia: the early archaeology of eastern and central Java. In Under the canopy: the archaeology of tropical rain forests (ed. J Mercader), pp. 161-190. Piscataway, NJ: Rutgers University Press.

[25] Sémah AM, Sémah F. 2012 The rain forest in Java through the Quaternary and its relationships with humans (adaptation, exploitation and impact on the forest). Quat. Int. **249**, 120-128. (10.1016/j.quaint.2011.06.013)

[26] Louys J, Roberts P. 2020 Environmental drivers of megafauna and hominin extinction in Southeast Asia. Nature **586**, 402-406. (10.1038/s41586-020-2810-y) 33029012

[27] Morwood MJ et al. 2004 Archaeology and age of a new hominin from Flores in eastern Indonesia. Nature **431**, 1087-1091. (10.1038/nature02956) 15510146

[28] Westaway KE et al. 2007 Age and biostratigraphic significance of the Punung rainforest fauna, east Java, Indonesia, and implications for *Pongo* and *Homo*. J. Hum. Evol. **53**, 709-717. (10.1016/j.jhevol.2007.06.002) 17706269

[29] Détroit F, Mijares AS, Corny J, Daver G, Zanolli C. 2019 A new species of *Homo* from the Late Pleistocene of the Philippines. Nature **568**, 181-186. (10.1038/s41586-019-1067-9) 30971845

[30] Hutterer KL. 1983 The natural and cultural history of Southeast Asian agriculture. Anthropos **78**, 169-212.

[31] Bailey R, Head G, Jenike M, Owen B, Rechtman R, Zechenter E. 1989 Hunting and gathering in tropical rain forest: is it possible? Am. Anthropol. **91**, 59-82. (10.1525/aa.1989.91.1.02a00040)

[32] Boyette AH, Lew-Levy S, Jang H, Kandza V. 2022 Social ties in the Congo Basin: insights into tropical forest adaptation from BaYaka and their neighbours. Phil. Trans. R. Soc. B **377**, 20200490. (10.1098/rstb.2020.0490) 35249385 PMC8899623

[33] Mercader J. 2000 Forest people: the role of African rainforests in human evolution and dispersal. Evol. Anthropol. **11**, 117-124. (10.1002/evan.10022)

[34] Westaway K et al. 2017 An early modern human presence in Sumatra 73,000–63,000 years ago. Nature **548**, 322-325. (10.1038/nature23452) 28792933

[35] Barker G et al. 2007 The ‘Human Revolution’ in lowland tropical Southeast Asia: the antiquity and behavior of anatomically modern humans at Niah Cave (Sarawak, Borneo). J. Hum. Evol. **52**, 243-261. (10.1016/j.jhevol.2006.08.011) 17161859

[36] Barker G, Farr L (eds) 2016 Archaeological investigations in the Niah Caves, Sarawak. The archaeology of the Niah Caves, Sarawak, Volume 2. Cambridge, UK: McDonald Institute for Archaeological Research.

[37] Deraniyagala SU. 1992 The prehistory of Sri Lanka: an ecological perspective, 2nd edn. Colombo, Sri Lanka: Department of Archaeological Survey.

[38] Roberts P, Perera N, Wedage O, Deraniyagala S, Perera J, Eregama S, Petraglia MD, Lee-Thorp JA. 2017 Fruits of the forest: human stable isotope ecology and rainforest adaptations in Late Pleistocene and Holocene (∼36 to 3 ka) Sri Lanka. J. Hum. Evol. **106**, 102-118. (10.1016/j.jhevol.2017.01.015) 28434535

[39] Wedage O et al. 2019 Specialized rainforest hunting by *Homo sapiens* ∼45,000 years ago. Nat. Commun. **10**, 739. (10.1038/s41467-019-08623-1) 30783099 PMC6381157

[40] Roberts P, Perera N, Wedage O, Deraniyagala S, Perera J, Eregama S, Gledhill A, Petraglia MD, Lee-Thorp JA. 2015 Direct evidence for human reliance on rainforest resources in Late Pleistocene Sri Lanka. Science **347**, 1246-1249. (10.1126/science.aaa1230) 25766234

[41] Roberts P, Stewart BA. 2018 Defining the ‘generalist specialist’ niche for Pleistocene *Homo sapiens*. Nat. Hum. Behav. **2**, 542-550. (10.1038/s41562-018-0394-4) 31209320

[42] Sullivan M et al. 2017 Diversity and carbon storage across the tropical forest biome. Sci. Rep. **7**, 39102. (10.1038/srep39102) 28094794 PMC5240619

[43] Cardoso AW et al. 2020 A distinct ecotonal tree community exists at central African forest–savanna transitions. J. Ecol. **109**, 1170-1183. (10.1111/1365-2745.13549)

[44] Crevecoeur I et al. 2014 First early hominin from Central Africa (Ishango, Democratic Republic of Congo). PLoS ONE **9**, e84652. (10.1371/journal.pone.0084652) 24427292 PMC3888414

[45] Shipton C et al. 2018 78,000-year-old record of Middle and Later Stone Age innovation in an East African tropical forest. Nat. Commun. **9**, 1832. (10.1038/s41467-018-04057-3) 29743572 PMC5943315

[46] Roberts P et al. 2020 Late Pleistocene to Holocene human palaoeecology in the tropical environments of coastal eastern Africa. Palaeogeog. Palaeroclimatol. Palaeoecol. **537**, 109438. (10.1016/j.palaeo.2019.109438)

[47] Scerri EML et al. 2021 Continuity of the Middle Stone Age into the Holocene. Sci. Rep. **11**, 7. (10.1038/s41598-020-78214-4) 33431997 PMC7801626

[48] Scerri EML et al. 2018 Did our species evolve in subdivided populations across Africa, and why does it matter? Trends Ecol. Evol. **33**, 582-594. (10.1016/j.tree.2018.05.005) 30007846 PMC6092560

[49] Braucher R, Oslisly R, Mesfin I, Ntoutoume PP. ASTER Team. 2022 *In situ*-produced ^10^Be and ^26^Al indirect dating of Elarmékora Earlier Stone Age artefacts: first attempt in a savannah forest mosaic in the middle Ogooué valley, Gabon. Phil. Trans. R. Soc. B **377**, 20200482. (10.1098/rstb.2020.0482) 35249387 PMC8899616

[50] Gosling WD, Scerri EML, Kaboth-Bahr S. 2022 The climate and vegetation backdrop to hominin evolution in Africa. Phil. Trans. R. Soc. B **377**, 20200483. (10.1098/rstb.2020.0483) 35249389 PMC8899624

[51] Kaboth-Bahr S et al. 2021 Paleo-ENSO influence on African environments and early modern humans. Proc. Natl Acad. Sci. USA **118**, e2018277118. (10.1073/pnas.2018277118) 34074756 PMC8201937

[52] Hublin JJ et al. 2017 New fossils from Jebel Irhoud, Morocco and the pan-African origin of *Homo sapiens*. Nature **546**, 289-292. (10.1038/nature22336) 28593953

[53] Taylor N. 2022 Riddles wrapped inside an enigma. Lupemban MSA technology as a rainforest adaptation: revisiting the lanceolate point. Phil. Trans. R. Soc. B **377**, 20200484. (10.1098/rstb.2020.0484) 35249391 PMC8899621

[54] Blinkhorn J, Timbrell L, Grove M, Scerri EML. 2022 Evaluating refugia in recent human evolution in Africa. Phil. Trans. R. Soc. B **377**, 20200485. (10.1098/rstb.2020.0485) 35249393 PMC8899617

[55] Stewart JR, Stringer CB. 2012 Human evolution out of Africa: the role of refugia and climate change. Science **335**, 1317-1321. (10.1126/science.1215627) 22422974

[56] Scerri EML, Blinkhorn J, Niang K, Bateman M, Groucutt HS. 2017 Persistence of Middle Stone Age technology to the Pleistocene/Holocene transition supports a complex hominin evolutionary scenario in West Africa. J. Archaeol. Sci. Rep. **11**, 639-646. (10.1016/j.jasrep.2017.01.003)

[57] Blome MW, Cohen AS, Tryon CA, Brooks AS, Russell J. 2012 The environmental context for the origins of modern human diversity: a synthesis of regional variability in African climate 150,000–30,000 years ago. J. Hum. Evol. **62**, 563-592. (10.1016/j.jhevol.2012.01.011) 22513381

[58] Orijemie EA. 2022 Human behaviour and climate-linked fluctuations in the rainforests of West-Central Africa. Phil. Trans. R. Soc B **377**, 20200488. (10.1098/rstb.2020.0488) 35249382 PMC8899626

[59] Blinkhorn J, Achyuthan H, Petraglia M, Ditchfield P. 2013 Middle Palaeolithic occupation in the Thar Desert during the Upper Pleistocene: the signature of a modern human exit out of Africa? Quat. Sci. Rev. **77**, 233-238. (10.1016/j.quascirev.2013.06.012)

[60] Scerri EML. 2017 The North African Middle Stone Age and its place in recent human evolution. Evol. Anthropol. **26**, 119-135. (10.1002/evan.21527) 28627786

[61] Groucutt HS et al. 2018 *Homo sapiens* in Arabia 85 thousand years ago. Nat. Ecol. Evol. **2**, 800-809. (10.1038/s41559-018-0518-2) 29632352 PMC5935238

[62] Corlett RT. 2014 The ecology of tropical east Asia. Oxford, UK: Oxford University Press.

[63] Roberts P et al. 2022 Fossils, fish and tropical forests: prehistoric human adaptations on the island frontiers of Oceania. Phil. Trans. R. Soc. B **377**, 20200495. (10.1098/rstb.2020.0495) 35249390 PMC8899615

[64] Perera N et al. 2011 People of the ancient rainforest: Late Pleistocene foragers at the Batadomba-lena rockshelter, Sri Lanka. J. Hum. Evol. **61**, 254-269. (10.1016/j.jhevol.2011.04.001) 21777951

[65] Liu W et al. 2015 The earliest unequivocally modern humans in southern China. Nature **526**, 696-700. (10.1038/nature15696) 26466566

[66] Langley MC et al. 2020 Bows and arrows and complex symbolic displays 48,000 years ago in the South Asian tropics. Sci. Adv. **6**, eaba3831. (10.1126/sciadv.aba3831) 32582854 PMC7292635

[67] Xhauflair H et al. 2016 Characterisation of the use-wear resulting from bamboo working and its importance to address the hypothesis of the existence of a bamboo industry in prehistoric Southeast Asia. Quatern. Int. **416**, 95-125. (10.1016/j.quaint.2015.11.007)

[68] Louys J et al. 2022 Speleological and environmental history of Lida Ajer cave, western Sumatra. Phil. Trans. R. Soc. B **377**, 20200494. (10.1098/rstb.2020.0494) 35249388 PMC8922409

[69] McAdams C, Morley MW, Fu X, Kandyba AV, Derevianko AP, Nguyen DT, Doi NG, Roberts RG. 2022 Late Pleistocene shell midden microstratigraphy indicates a complex history of human–environment interactions in the uplands of North Vietnam. Phil. Trans. R. Soc. B **377**, 20200493. (10.1098/rstb.2020.0493) 35249386 PMC8899622

[70] Braje TJ, Dillehay TD, Erlandson JM, Klein RG, Rick TC. 2017 Finding the first Americans. Science **358**, 592-594. (10.1126/science.aao5473) 29097536

[71] Dillehay TD. 2017 Where the land meets the sea: fourteen millennia of human history at Huaca Prieta, Peru. Austin, TX: University of Texas Press.

[72] Pedersen MW et al. 2016 Postglacial viability and colonization in North America's ice-free corridor. Nature **537**, 45-49. (10.1038/nature19085) 27509852

[73] Lothrop SK. 1961 Early migrations to Central and South America: an anthropological problem in the light of other sciences. J. R. Anthropol. Inst. G. B. Ireland **91**, 97-123.

[74] Lynch TF. 1990 Glacial-age man in South America? A critical review. Am. Antiq. **55**, 12-36. (10.2307/281490)

[75] Sauer CO. 1944 A geographic sketch of early man in America. Geogr. Rev. **34**, 529-573. (10.2307/210028)

[76] Aceituno FJ, Loaiza N. 2018 The origins and early development of plant food production and farming in Colombian tropical forests. J. Anthropol. Archaeol. **49**, 161-172. (10.1016/j.jaa.2017.12.007)

[77] Iriarte J. 2007 New perspectives on plant domestication and the development of agriculture in the New World. In Rethinking agriculture: archaeological and ethnoarchaeological perspectives (eds TP Denham, J Iriarte, L Vyrdaghs), pp. 165-186. Wallnut Creek, CA: Left Coast Press.

[78] Lombardo U, Iriarte J, Hilbert L, Ruiz-Perez J, Capriles JM, Veit H. 2020 Early Holocene crop cultivation and landscape modification in Amazonia. Nature **581**, 190-193. (10.1038/s41586-020-2162-7) 32404996 PMC7250647

[79] Morcote-Ríos G, Aceituno FJ, Leon Sicard TIR, Antes de S, Orellana P. 2014 Recolectores del Holoceno Temprano en la Floresta Amazonica Colombiana. In Actas del 3er Encuentro Internacional de ArqueologíaAmazonica (ed. S. Rostain), pp. 39-50. Quito, Ecuador: Instituto Frances de Estudios Andinos.

[80] Piperno DR. 2011 The origins of plant cultivation and domestication in the New World tropics: patterns, process, and new developments. Curr. Anthropol. **52**, S453-S470. (10.1086/659998)

[81] Roosevelt AC et al. 1996 Paleoindian cave dwellers in the Amazon: the peopling of the Americas. Science **272**, 373-384. (10.1126/science.272.5260.373)

[82] Shock MP, Moraes CdP. 2019 A floresta e o domus: a importancia das evidencias arqueobotanicas e arqueologicas das ocupaçoes humanas amazonicas na transiçao Pleistoceno/Holoceno. Boletim do Museu Paraense Emílio Goeldi. Ciencias Humanas **14**, 263-289.

[83] Maezumi SY et al. 2018 The legacy of 4,500 years of polyculture agroforestry in the eastern Amazon. Nat. Plants **4**, 540-547. (10.1038/s41477-018-0205-y) 30038410 PMC6119467

[84] Levis C et al. 2017 Persistent effects of pre-Columbian plant domestication on Amazonian forest composition. Science **355**, 925-931. (10.1126/science.aal0157) 28254935

[85] Iriarte J, Elliott S, Maezumi SY, Alves D, Gonda R, Robinson M, Gregorio de Souza J, Watling J, Handley J. 2020 The origins of Amazonian landscapes: plant cultivation, domestication and the spread of food production in tropical South America. Quat. Sci. Rev. **248**, 106582. (10.1016/j.quascirev.2020.106582)

[86] Sales RK, McMichael CNH, Flantua SGA, Hagemans K, Zondervan JR, González-Arango C, Church WB, Bush MB. 2022 Potential distributions of pre-Columbian people in Tropical Andean landscapes. Phil. Trans. R. Soc. B **377**, 20200502. (10.1098/rstb.2020.0502) 35249384 PMC8899625

[87] Malhi Y, Doughty CE, Galetti M, Smith FA, Svenning JC, Terborgh JW. 2016 Megafauna and ecosystem function from the Pleistocene to the Anthropocene. Proc. Natl Acad. Sci. USA **113**, 838-846. (10.1073/pnas.1502540113) 26811442 PMC4743772

[88] Anderman T, Faurby S, Turvey ST, Antonelli A, Silvestro D. 2020 The past and future human impact on mammalian diversity. Science **6**, eabb2313. (10.1126/sciadv.abb2313) PMC7473673 32917612

[89] Iriarte J, Ziegler MJ, Outram AK, Robinson M, Roberts P, Aceituno FJ, Morcote-Ríos G, Keesey TM. 2022 Ice Age megafauna rock art in the Colombian Amazon? Phil. Trans. R. Soc. B **377**, 20200496. (10.1098/rstb.2020.0496) 35249392 PMC8899627

[90] Robinson M, Morcote-Rios G, Aceituno FJ, Roberts P, Berrio C, Iriarte J. 2021 ‘Moving South’: Late Pleistocene plant exploitation and the importance of palm in the Colombian Amazon. Quaternary **4**, 26. (10.3390/quat4030026)

[91] Watling J, Shock MP, Mongeló GZ, Almeida FO, Kater T, De Oliveira PE, Neves EG. 2018 Direct archaeological evidence for Southwestern Amazonia as an early plant domestication and food production centre. PLoS ONE **13**, e0199868. (10.1371/journal.pone.0199868) 30044799 PMC6059402

[92] Grove M. 2015 Palaeoclimates, plasticity, and the early dispersal of *Homo sapiens*. Quatern. Int. **369**, 17-37. (10.1016/j.quaint.2014.08.019)

[93] McMichael CN. 2020 Ecological legacies of past human activities in Amazonian forests. New Phytol. **229**, 2492-2496. (10.1111/nph.16888) 32815167 PMC7891632

[94] Caetano-Andrade VL, Clement CR, Weigel D, Trumbore S, Boivin N, Schöngart J, Roberts P. 2020 Tropical trees as time capsules of anthropogenic activity. Trends Plant Sci. **25**, 369-380. (10.1016/j.tplants.2019.12.010) 32037081

[95] Xhauflair H, Pawlik A, Forestier H, Saos T, Dizon E, Gaillard C. 2017 Use-related or contamination? Residue and use-wear mapping on stone tools used for experimental processing of plants from Southeast Asia. Quat. Int. **427**, 80-93. (10.1016/j.quaint.2016.02.023)

[96] Reynolds TEG. 1990 The Hoabinhian: a review. In Bibliographic review of Far Eastern archaeology (ed. GL Barnes), pp. 1-30. Oxford, UK: Oxbow Books.

